# On‐chip technology for single‐cell arraying, electrorotation‐based analysis and selective release

**DOI:** 10.1002/elps.201900097

**Published:** 2019-06-03

**Authors:** Kevin Keim, Mohamed Z. Rashed, Samuel C. Kilchenmann, Aurélien Delattre, António F. Gonçalves, Paul Éry, Carlotta Guiducci

**Affiliations:** ^1^ Laboratory of Life Sciences Electronics École Polytechnique Fédérale de Lausanne Lausanne Switzerland

**Keywords:** Dielectrophoretic trapping, Electrorotation, Single‐cell analysis, Single‐cell array, Single‐cell release

## Abstract

This paper reports a method for label‐free single‐cell biophysical analysis of multiple cells trapped in suspension by electrokinetic forces. Tri‐dimensional pillar electrodes arranged along the width of a microfluidic chamber define actuators for single cell trapping and selective release by electrokinetic force. Moreover, a rotation can be induced on the cell in combination with a negative DEP force to retain the cell against the flow. The measurement of the rotation speed of the cell as a function of the electric field frequency define an electrorotation spectrum that allows to study the dielectric properties of the cell. The system presented here shows for the first time the simultaneous electrorotation analysis of multiple single cells in separate micro cages that can be selectively addressed to trap and/or release the cells. Chips with 39 micro‐actuators of different interelectrode distance were fabricated to study cells with different sizes. The extracted dielectric properties of Henrietta Lacks, human embryonic kidney 293, and human immortalized T lymphocytes cells were found in agreements with previous findings. Moreover, the membrane capacitance of M17 neuroblastoma cells was investigated and found to fall in in the range of 7.49 ± 0.39 mF/m^2^.

**Abbreviations**
CM: Clausius‐Mossotti
DEP: dielectrophoresis
HEK 293: human embryonic kidney 293
HeLa: Henrietta Lacks
nDep: negative dielectrophoresis
PCB: printed circuit board
ROT: electrorotation
SU‐8: Gersteltec GM 1070
Ti: titanium

## 1 Introduction

Electrorotation (ROT) is a label‐free analysis technique 1, 2, which can read out the dielectric properties of cells. It can differentiate between cell lines 3, observe cell membrane changes 4, 5, 6, or investigate the cytoplasm properties of cells 7, to only mention some examples.

**Figure 1:**
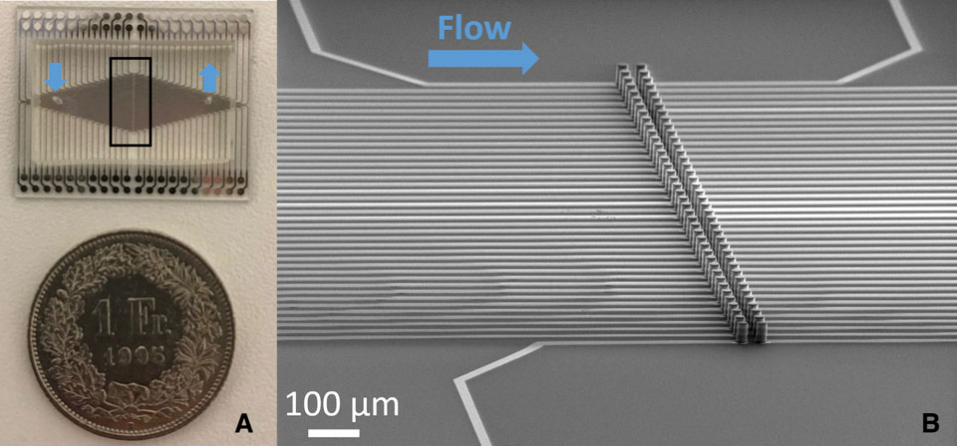
(A) Photograph of a bonded chip with a width of the microfluidic channel of 6.4 mm and with both electrode diameter and interelectrode distance of 80 µm. The black box shows the region observed by scanning electron microscopy shown in (B). (B) Scanning electron microscope micrograph of two arrays of metal covered SU‐8 electrodes, which are separately connected by an underlying, insulated metal wire. The electrode´s diameters and interelectrode distance is 20 µm.

Existing systems employing this technique suffer from a very low parallel operation. Simultaneous rotation of multiple single cells in an array has never been demonstrated so far. The technologies used to integrate electrorotation actuators on microfluidic chips allows a very limited scalability and flexibility in the channel height and cage size. We apply the vertical electrodes integration approach that we presented in previous works 8, 9 to develop a system for single cells arraying and parallel electrorotation analysis in 50 µm high microfluidic chambers (Fig. 1B). Each electrode is connected to a dedicated pad to allow the independent control of each single‐cell actuator to capture a cell, retain it, rotate it by electric field of varying frequencies while being held against the flow, and finally selectively released.

Dielectrophoretic forces have been used previously to arrange cells in arrays. For instance, negative dielectrophoretic (nDEP) traps can capture cells at electric field minima 10, 11. In a device available on the market by Silicon Biosystems, multiple cells can be individually positioned in a 2D array to be analyzed by fluorescence through optical markers 11. However, after positioning, the cells are closed into the chamber and no flow is applied. Devices to trap and release cells in flow, arrayed in a selective manner, have been developed using pillar‐like electrodes, exploiting the higher efficiency of 3D structures to apply dielectrophoretic holding forces 10, 12.

3D microelectrodes have been fabricated by additive techniques, such as electroplating 13, 14, 15, 16, pyrolysis of photoresist 17, 18, or ion implantation into PDMS 19. Subtractive techniques have also been employed such as based on etching of highly doped silicon 20, 21, or on the conformal deposition of conductive materials on a 3D scaffold and their subsequent pattering 22, 23. Our group has recently developed a novel fabrication method to obtain high aspect ratio of tri‐dimensional electrodes of any desired shape by covering passive negative photoresist Gersteltec GM 1070 (SU‐8) cores with metal and subsequent mask‐less etching which only lets metal to remain on the sidewall of the cores 8. This technique combines the advantages of both additive and subtractive techniques, it is fast and inexpensive compared to electroplating, has high conductive electrodes compared to pyrolysis technique, doped silicon and ion implanted PDMS, as well as a micrometer precision.

Previous systems used an asymmetric electrode configuration and electric signals of the same amplitude to create open dielectrophoretic cages upstream to trap cells and analyze them by fluorescence 10. In our system, we can address the four electrodes of the quadrupole separately, thus control the entry of the cells then retain them by blocking additional cells from entering the same trap. The first advantage of this configuration is that the same electrodes can be used for two neighboring traps, which enables to control the entire section of the flow chamber along the array of traps. This feature is crucial for handling low concentration of cells of interest inside the flow.

The second advantage is that the quadrupole is composed by four electrodes arranged symmetrically and close to each other, which grants high quality electrorotation spectra. In order to acquire accurate electrorotation spectra, cells have to be in the center of the rotating electric field. In large electrorotation chambers laser tweezers have been used in order to center the cells and keep them in place 24, 25, 26, so the cell experiences the torque corresponding to its position within the electrodes throughout the measurement 27. This clearly comes at the cost of scalability and complexity provided by such techniques. Other approaches alternate nDEP signals and ROT actuation 28 or superpose them 3, 29. Octopoles, which consist of a quadrupole placed on the bottom of a channel and one at the top 30, can create a rotating electric field, by placing the top and bottom quadrupole rotated by an angle 31 and perform electrorotation experiments.

All these systems report one single cage except for the work by Fuhr et al. where three single‐cell octopoles are placed after each other in a narrow microfluidic channel 25. However, simultaneous electrorotation on multiple single cells were not reported.

In our system, multiple trapping and rotation of single cells is achieved by independently‐addressed single quadrupole cages positioned perpendicularly to the flow stream in a large microfluidic chamber.

## 2 Materials and methods

### 2.1 Experimental

#### 2.1.1 Microfabrication

The dielectrophoretic microcages consist of two arrays of 40 3D electrodes integrated within a microfluidic channel. This leads to a total amount of 39 arrayed micro cages. The height of the electrodes and of the surrounding microfluidic channel is 50 µm. The diameter of the electrodes and of the inter electrode distance varies between 20, 40, and 80 µm.

First, a Ti/Pt/Ti (20/200/20 nm) is sputtered (*Pfeiffer Spider 600*) on the plane wafer and connection lines are subsequently patterned by ion beam etching (*Veeco Nexus IBE350*). Then, a SiO_2_ (300 nm) layer is sputtered to insulate the wires. Vias (*SPTS APS Dielectric Etcher*) are etched into the insulation layer where the 3D electrodes and the contacts from the printed circuit board (PCB) to the chip are placed later on. A 50 µm SU‐8 skeleton is patterned on the uninsulated metal. The whole wafer and therefore the SU‐8 skeleton as well is covered with a Ti/Pt (20 nm/200 nm) metal layer by sputtering. This process achieves the coating of the side walls of the SU‐8 skeleton. The metal on the planar wafer and on top of the electrodes is subsequently removed by ion beam etching without an etching mask. Due to an etching angle of 0° perpendicular to the wafer surface, the metal on the side walls of the SU‐8 skeleton remains. This process was presented in 8 except for the insulation of the planar wires with sputtered SiO_2_. A second layer of 50 µm SU‐8 is coated and patterned on the wafer, forming the wide microfluidic channel. The chip is closed by irreversibly bonding PDMS to the SU‐8 channel 32. A photograph of the final chip is shown in Fig. 1A.

#### 2.1.2 Cell preparation

Henrietta Lacks (HeLa), human embryonic kidney 293 (HEK 293), and BE(2)‐M17 human neuroblastoma cells are cultured in *DMEM*, human immortalized T lymphocytes are grown in suspension in Roswell Park Memorial Institute Medium. In both media, 10% Fetal bovine serum and 1% antibiotics (L‐Glutamine‐penicillin‐streptomycin) are added. Before the experiments, the adherent HeLa, HEK 293 and M17 cells are detached from the surface of the culture flask using 1X *Trypsin* and all cells are resuspended in an isotonic solution (8.6% dextrose and 0.3% sucrose) with an adapted conductivity of 100 mS/m using 1X PBS. The cell concentration was around 200 000 cells per milliliter.

Before the experiments, the chips are primed by flushing 2 mL of the corresponding culture medium (including Fetal bovine serum) and subsequently by flushing 2 mL of the 100 mS/m solution, in which the cells are suspended. In many examples, electrorotation experiments are performed at a medium conductivity of 56 mS/m, which is found to be the conductivity at which the cytoplasm conductivity of cells can be investigated 7, 33. However, in order to obtain nDEP at frequencies high enough to avoid electrolysis at the electrodes, 100 mS/m conductive media was used.

#### 2.1.3 Measurement procedure

Cells are injected in the microchips with the proper electrode diameter size based on cells size. HeLa, HEK 293, and M17 cells are injected in chips with an electrode diameter of 40 and 80 µm. They are not injected in chips with an interelectrode distance of 20 µm, since they occasionally cause clogging due to their size, especially if cell clusters are formed. T lymphocytes can be injected in chips featuring any of the three electrode diameter.

**Figure 2:**
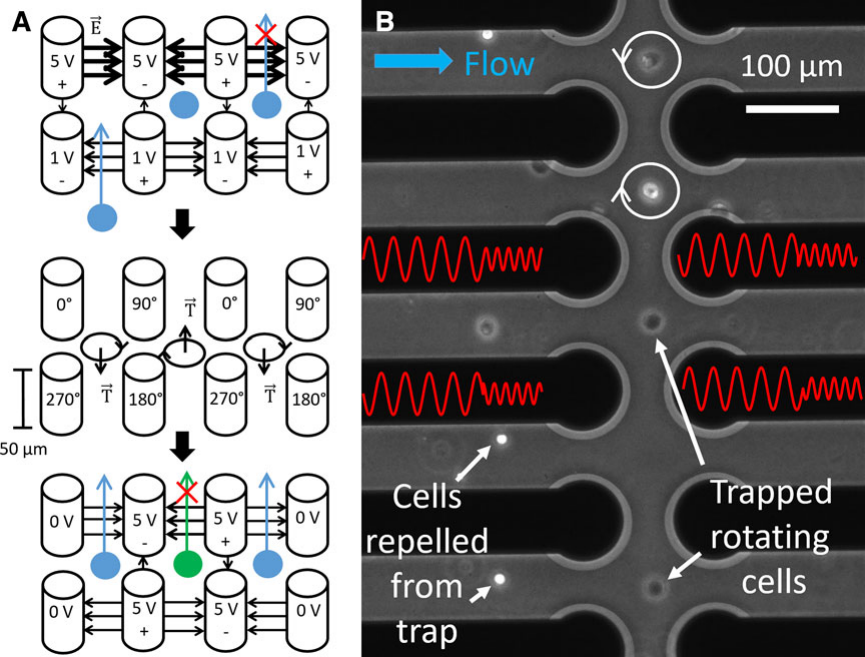
(A) Working principle of the microcage array. Single cells are trapped, analyzed by electrorotation and are selectively released. (B) Microscope image of rotating cells simultaneously trapped within the microcage array by alternating DEP and ROT signals. The first DEP signal has a phase shift of 180° between neighboring electrodes and exerts a trapping force. The second ROT signal has a phase shift of 90° between neighboring electrodes (the electric signals on the electrodes are illustrated in red) and exerts a torque on the cells making them rotate. Cells outside the cages are repelled and this cannot enter due to the presence of a dielectrophoretic barrier.

The cells in suspension are driven through the device by a flow‐rate between 200 and 1 µL/min. With no electric field applied, they simply pass by the upstream (entrance) electrodes first and then the downstream (exit) electrodes. For the trapping of cells, the arbitrary waveform generator (*TTi TGA12104*) creates four signals. Two signals of 5 V amplitude and a phase shift of 180° and two signals of 1 V amplitude and a phase shift of 180°. Applying an alternating current (100 kHz) electric voltage signal of 1 V amplitude at the entrance and of 5 V amplitude at the exit electrodes creates a lower dielectrophoretic barrier at the entrance and a higher barrier at the exit of the array. Due to the drag force of the fluid flow, the cells can pass the lower entrance barrier, but cannot overcome the higher exit barrier, consequently they are trapped in the array, as shown on the left sketch of Fig. 2(A).

After a cell is trapped in a single‐cell microcage, the voltage at the two upstream electrodes is raised to 5 V amplitude preventing additional cells to enter the trap (the voltage is changed by means of selection circuitry on the PCB, described in the Supporting Information chapter 3). The other traps remain open, with an entrance voltage of 1 V amplitude. Adjacent quadrupoles now have one entrance electrode with an applied voltage of 1 V amplitude and one with 5 V amplitude, however, the dielectrophoretic barrier is still low enough to allow incoming cells to enter the trap. After a sufficient amount of filled single‐cell traps is reached, all traps are closed and the electric signal is alternated between a DEP trapping (5 V amplitude, 100 kHz and a phase shift of 180° between neighboring electrodes) and a ROT signal (2.5 V amplitude, a swept frequency and 90° phase shift between neighboring electrodes) as shown in Fig. 2B. A similar approach was already presented by Rohani et al. on planar electrodes 28. The ROT signal generates a torque on the cells and causes them to rotate (Fig. 2A middle). Cells in neighboring microcages rotate in the opposite direction, since the two electrodes of the rotation quadrupole are shared. A microscope image of the measurement in which four single cells are rotating within the array and another four cells are prevented from entering the trap is shown in Fig. 2B and Supporting Information Video 1. The frequency of the rotating electric field is swept between 10 kHz and 10 MHz in 25 logarithmic steps, the switching of the frequency can be seen in Supporting Information Video 2. Multiple single cells are rotating simultaneously in individual neighboring microcages, as shown in Supporting Information Video 4. Videos of the cells rotation are acquired with a frame rate between 5 and 25 Hz with a microscope camera (*Andor Neo sCMOS*) for 3 s for each electric signal frequency. The rotation speed of the cells rotating in the videos were acquired by an automatic pattern matching algorithm implemented in LabVIEW. Since T lymphocytes could not get held against the flow by cages of 40 and 80 µm interelectrode distance, the flow was stopped as soon as the T lymphocytes were within the microcage array. Hence, the nDEP force was sufficient to center them and their electrorotation spectra could be acquired, as shown in Supporting Information Video 3 and 4.

After the acquisition of the ROT spectra, the cells can be selectively released by turning off the electric signal at one of the exit electrodes. The exit barrier is thus reduced and the cell is carried away by the flow as sketched at the bottom of Fig. 2A.

If the chips are clogged with organic material like cells, cell fragments, or bacteria, they can be cleaned by flushing 1% sodium hypochlorite through the chip until all organic contamination are flushed away. Subsequently, the chip is flushed with culture medium and 100 mS/m solution in order to remove sodium hypochlorite residuals. Using this procedure, the chips could be used for several weeks during continuous experiments.

### 2.2 Dielectrophoretic force and electrorotation phenomenon

The dielectrophoretic force **F**_DEP_, which balances the drag force of the fluid flow when the cell is stably trapped in the center of the microcage, is given by 34

(1) $$〈F_{D}EP〉=\pi \varepsilon_{0}\varepsilon_{m}R^{3}Re\left[CM\right]\nabla \;E_{pk}^{2}$$

Here, ε_0_ is the absolute and ε_m_ is the relative permittivity of the medium; *R* is the cell radius and Re[*CM*] is the real part of the Clausius‐Mossotti factor, which depends on the dielectric properties of the cell and the surrounding medium. ∇$\;E_{pk}^{2}$ is the gradient of the electric field amplitude.

The speed of electrokinetically‐induced rotation is 34

(2) Ω=−ε0εm2η Im CME2

with the medium viscosity η and Im[*CM*] the imaginary part of the Clausius‐Mossotti factor. The Clausius‐Mossotti factor's expression depends on the model used for the cell and is given by 35

(3) CM=ε∼p−ε∼mε∼p+2ε∼m;ε∼p=C∼ mem 3Rε∼ cyto 3ε∼ cyto +3C∼ mem R

${\tilde{\varepsilon }}_{p}$, ${\tilde{\varepsilon }}_{m}$, ${\tilde{\varepsilon }}_{cyto}$are the complex permittivity of the particle, the suspending medium and the cytoplasm respectively, defined as: $\tilde{\varepsilon }$ =ε– iσ/ω and ${\tilde{C}}_{mem}$ is the complex membrane capacitance defined as $\tilde{C}$ = C – i*G*/ω with *G* the membrane conductance.

### 2.3 Simulations

**Figure 3:**
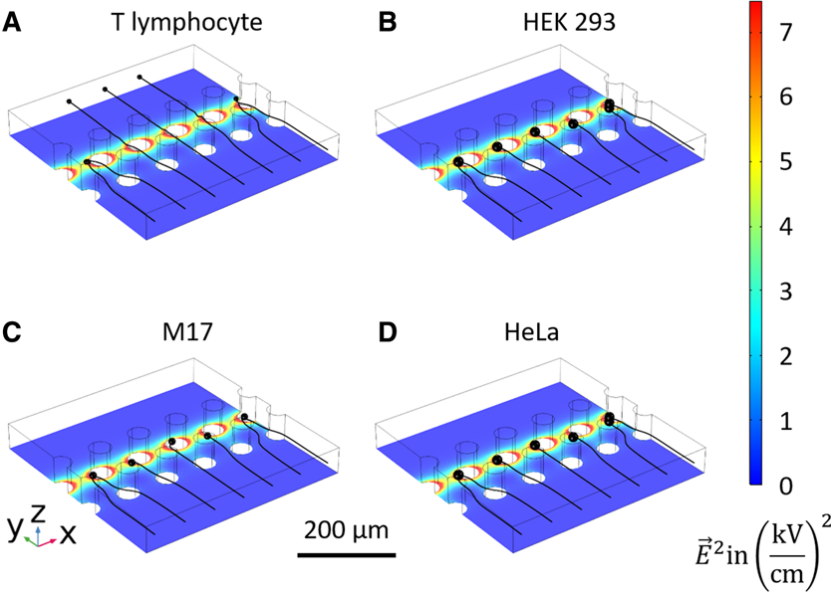
Finite element simulations of (A) human T lymphocytes, (B) HEK 293 cells, (C) M17 neuroblastoma and (D) HeLa cells in a microfluidic channel with an applied electric field of 5 V amplitude at the exit and 1 V amplitude at the entrance electrodes. The cells with a larger diameter (HEK 293 cells, M17 neuroblastoma and HeLa) (B–D) are trapped between the two electrode arrays with an interelectrode distance of 40 µm, while the T lymphocytes (A) are not. The square of the electric field is illustrated by a rainbow color coding. The particle's trajectory is shown in black.

Finite element simulations of the trap configurations are performed using COMSOL Multiphysics^®^. The electrode configurations as described in the microfabrication section are implemented in a cuboid of 400 µm per 400 µm per 50 µm. The flow in the device is simulated using laminar flow. The pressure difference between the inlet and the outlet is varied logarithmically between 0.001 and 0.1 mbar. The electric field is simulated using the electric current module. At the entrance electrodes an electric potential of +1 V and ‐1 V is applied and at the exit an electric potential of +5 V and ‐5 V is applied. The absolute value of the electric field is illustrated in the background color of the simulations shown in Fig. 3. In order to see if a specific cell type is getting trapped in the electrode configuration, we used the COMSOL^®^ module for particle tracing in fluid flow. Particles with the cells' diameters are created at the high‐pressure side of the cuboid. The drag force on the particles, as well as a self‐implemented DEP force based on Eq. (1) are calculated from the laminar flow profile and the electric field. The particle trajectory of T lymphocytes (a), HEK 293 cells (b), M17 cells (c) and HeLa cells (d) in the 3D space for an interelectrode distance of 40 µm is calculated and illustrated in Fig. 3. The simulations for interelectrode distances of 20 µm and of 80 µm can be found in the Supporting Information (chapter 2).

In the nDEP regime at a frequency of 100 kHz, three trapping scenarios are possible depending on the microfluidic pressure and the trap size. First, the pressure is not sufficient and the cells do not overcome the entrance barrier (as shown in the Supporting Information chapter 2 for HEK 293, M17 and HeLa cells in an array of an interelectrode distance of 20 µm). Second, the cells are being trapped in the array, as shown in Fig. 3B–D and, third, the cells go through the array and are not trapped, as shown in Fig. 3 A for T lymphocytes. The DEP trapping force depends on the cell type. HEK 293, M17 as well as HeLa cells can get retained against the flow in cages with interelectrode distance of 40 µm and 80 µm, as opposed to T lymphocytes. However, the latter are being trapped in cages with 20 µm interelectrode distance (See Supporting Information). On the other hand, for the same electrode configuration and pressure difference, HEK 293, M17, and HeLa cells are not entering the nDEP trap, but are blocked by the entry barrier. A more detailed description of the simulations models and methods used here can be found in 36.

## 3 Results and discussion

Cells of four different cell lines are injected and driven through the chips as described in the measurement procedure. We could trap four different cell types in our arrays controlling the entry of cells in individual cages. Multiple electrorotation spectra of different cell types were acquired. Finally, single cells were selectively released by lowering the dielectric barrier at the downstream electrodes.

### 3.1 Electrode diameter and trapping outcome

HeLa, HEK 293 cells, and M17 neuroblastoma are getting trapped in arrays with 40 µm and 80 µm inter electrode distance, while human immortalized T lymphocytes are not, which confirms the simulated expectations. A possible explanation for this could be the smaller cell radius *R* of such cells, which enters cubic into the DEP force. The force might not be large enough to overcome the fluid drag force. Reducing the interelectrode distance to 20 µm, not only the electric field becomes stronger, but as well its gradient ∇$E_{pk}^{2}$. This leads to a major increase of the trapping force and enable the trapping of the smaller T lymphocytes in the 20 µm interelectrode distance arrays. One of the main advantages of the presented electrode configuration vs. planar or octopole‐based solutions is that it generates a homogeneous electric field over the complete channel height and, therefore, equivalent DEP force. The presented 3D electrodes generate efficiently a holding force in the middle of the channel, where the drag force is the strongest, due to the parabolic flow profile.

### 3.2 Electrorotation spectra of single cells

**Figure 4:**
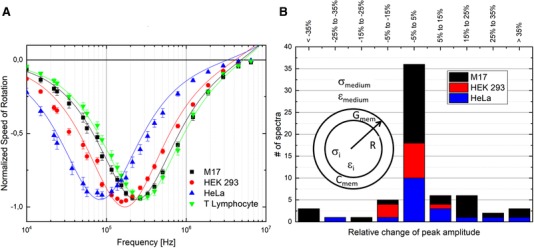
(A) Averaged and normalized electrorotation spectra and standard error of M17 neuroblastoma cells (black), HEK 293 (red) cells, HeLa (blue) cells, and human immortalized T lymphocytes (green) and their corresponding theoretical curves based on the extracted cell parameters. (B) Evaluation of the relative change of the absolute peak amplitude before and after the exposure to the rotating electric field for 5 min.

Using the protocol described in the measurement procedure, electrorotation spectra of 20 immortalized human T lymphocytes, 33 HEK 293 cells, 14 HeLa cells, and 29 M17 neuroblastoma were acquired. In total, over 140 single‐cell spectra were recorded, including repeated measurements on the same cell to characterize the stability of the system. The average electrorotation spectra and its standard error of each population are shown in Fig. 4A. The spectra were normalized by dividing the speed of rotation in each point by the corresponding speed of rotation of the negative peak of each spectrum. The curves based on the extracted parameters are traced as a continuous line. The first acquired electrorotation spectrum of each cell was fitted to the single shell model, depicted in Fig. 4B, using a least square method. The cell radii were measured based on the optical images on the chip. Since our measurements were performed for frequencies below 10 MHz, the cytoplasm parameters hardly influence the electrorotation spectrum 35, and were therefore fixed to values reported in agreement with the literature. For T lymphocytes 3, 6, 37, 38, 39 and HEK 293 cells 40, 41, 42, 43 a cytoplasm conductivity of 0.5 S/m and a cytoplasm permittivity of 78ε_0_ (same as water) are assumed. M17 neuroblastoma are fitted with these cytoplasm parameters too, since these are values commonly used for several cell lines. For HeLa cells a cytoplasm conductivity of 0.84 S/m was chosen as well as a cytoplasm permittivity of 60ε_0_ 44, 45. The membrane conductance was fixed to a value of 100 S/m^2^ for T lymphocytes 38, 39 and 0.95 S/m^2^ for HeLa cells 44. For HEK 293 cells and M17 neuroblastoma the membrane conductance was kept floating. However, this parameter should have little influence on the electrorotation spectrum 35.

Consecutive acquisitions of a spectrum of a single HEK 293 cell every 5 min over 30 min time period were performed (Supporting Information chapter 1). The difference between the peak frequency overall was ±10%, which is lower than what was reported when using laser tweezers (approx. 50% in 40 min) 25. In order to investigate the stability of the acquired spectra over time, electrorotation of 63 cells was performed before and after 5 min exposure to nDEP trapping and rotation within the array. The relative change of the peak amplitude is illustrated in Fig. 4B. 36 out of 63 cells experience a variation of less than ±5%, the other vary more. A possible explanation for the variation of the peak amplitude could be that the cells are observed in flow and possibly particulates in the solution or fluctuations within the solution might impact the rotation. However, the different contributions to this variation, on one side the sources of noise derived by the unprecise definition of the cell position and, on the other side, the actual changes of the rotation speed over time has to be further investigated.

The extracted membrane conductance data reported in Table 1 are in agreement with the literature (immortalized human T lymphocytes 3, 6, 37, 38, 39; HEK 293 cells 40, 41, 42, 43 and HeLa cells 44, 45). This demonstrates the functionality and accuracy of the proposed system. However, the specific membrane capacitance of M17 neuroblastoma has, to our knowledge, not yet been reported in the literature. Hence, we could not compare the value we extracted, i.e., 7.49 ± 0.39 mF/m^2^, to any previous work.

**Table 1:** The extracted dielectric parameters and their standard error compared to the values found in literature. The values indicated with * were fixed. The cell radii were measured by optical observation of the cells in suspension

Cell Type	Radius (µm) (measured)	Cytoplasm conductivity σ_i_ (S/m)	σ_i_ reported in literature (S/m)	Cytoplasm permittivity ε_i_	ε_i_ reported in literature	Membrane Conductance G_mem_ (S/m^2^)	G_mem_ reported in literature (S/m^2^)	Specific Membrane Capacitance C_mem_ (mF/m^2^)	C_mem_ reported in literature (mF/m^2^)	(Ref)
Human T‐lymphocyte (*n*=20)	4.75 ± 0.12	0.5*	0.5	78*	78	100*	10–1000	8.05 ± 0.47	13.49 ± 4.72	3
			0.53 ± 0.1		100		‐		7.01 ± 0.91	6
			0.65 ± 0.15		103.9 ± 24.5		‐		10.5 ± 3.1	38
			1.06 ± 0.14		74.0 ± 5.3		100		12.1 ± 1.4	39
			0.3–1		85		‐		11±1.1	40
HEK293 (*n*=33)	6.75 ± 0.5	0.5*	0.175 ± 0.014	78*	85 ± 15	3∙10^‐14^	0	9.81 ± 0.39	7.5 ± 0.3	41
			‐		‐		‐		11.1 ± 0.8	42
			0.5		60		7 ∙ 10^‐14^		‐	43
			0.408 ± 0.019		85 ± 4		≈0		7.94 ± 0.4	44
Hela (*n*=14)	8.74 ± 1.33	0.84*	‐	60*	47	0.95*	0.95‐1.2	17.51 ± 0.75	18.5 ± 2.6	45
			0.435–1.25		35–60		‐		19	46
M17 (*n*=29)	6.30 ± 1.29	0.5*	‐	78*	‐	5∙10^‐13^	‐	7.49 ± 0.39	‐	

The number of cells possible to analyze at once is limited by the field of view of the used microscope. Using a 20X magnification with a field of view of 690 µm * 582 µm, we could observe 10 quadrupoles with 40 µm interelectrode distance could be acquired simultaneously. Moreover, using a magnification of 10X and arrays with an inter electrode distance of 20 µm, all 39 traps could be observed. The image quality would need to be further improved in order to acquire spectra with such low magnification. Filters could help improving the contrast 46. Another possibility could be to use an automatized microscope stage 47 to observe all 39 quadrupoles with a 20X magnification in sequential videos.

At a cell concentration of 200 cells/ml and a flow rate of 1 µl/min, a new cell arrives every 12 s at a single trap, which leads to a total batch process time of about 90 s, including the spectra acquisition time. Including the statistical probability that some traps remain empty or occupied by multiple cells, this leads to a throughput of about 600 cells/h. Electrorotation systems are usually low throughput and therefore many papers do not mention this property explicitly. However, using the combination of a laser tweezer and electrorotation 26, a spectrum of a single cell is acquired in less than 3 min and the laser tweezer is applied for less than 5 min, which leads to an estimated throughput of 12 to 20 cells/h. Our system has a potential throughput, that we estimated to be 30 to 50 times higher.

### 3.3 Single‐cell release

**Figure 5:**
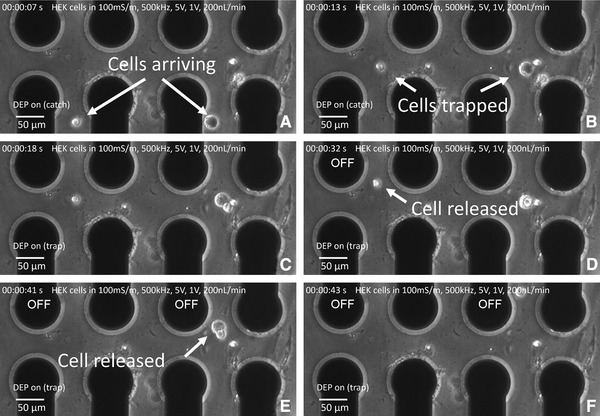
(A–D) Image series of the release mechanism. (A–C) two HEK 293 cells are trapped simultaneously in the microcage array. (E) Removing the electric signal on the very left electrode releases the left cell. (F), (G) Removing the signal on the electrode bordering the cell on the right releases it.

After the cells are trapped and analyzed, they can be released selectively. The chip design with separate interconnections for every electrode allows to change the signal applied by a single or several electrodes. Every interconnection on the chip is polarized by a dedicated pin to a PCB, where multiplexers direct the four signals supplied by the frequency generator to the corresponding electrodes. In order to release cells selectively, the signal of an exit electrode of the microcage is set to 0V. An experimental illustration of the release mechanism is shown in Fig. 5. The ability to turn off the electric signal of a single electrode using the switches on the PCB allows to turn off the retaining action of a microcage in which a cell is trapped. In Fig. 5 (A–C) two cells are getting trapped in the array. In Fig. 5D the electric signal on the left electrode is turned off and the left cell is released. In Fig. 5E the electric signal of the right electrode is turned off and the cell on the right is released too, as shown in Supporting Information Video 5.

## 4 Concluding Remarks

In this paper, we present a full system for simultaneous label‐free analysis of multiple single cell dielectric properties. Electrorotation spectra of arrayed single cells are acquired multiple while the cells are held against the flow in a wide microfluidic channel to monitor cells over time. Fabricated arrays with different cage size were designed to achieve efficient cell retention that facilitated the acquisition of more than a hundred spectra of single cells from four different cell types. The populations can be clearly differentiated and the extracted membrane capacitance for HeLa, HEK 293, and human immortalized T lymphocytes are in agreement with the values previously reported. Moreover, the dielectric properties of M17 neuroblastoma cells were characterized and reported for the first time by using electrokinetic based technique.

Currently we integrated 39 micro cages in our system, but could only observe 10 micro cages at a time in sufficient image resolution to acquire the spectra. Improving the imaging quality, decreasing the size of the cages and employing large‐scale optical observation system could make it possible to observe many arrays on the same chip simultaneously. The microfluidic system itself is able to acquire spectra of non‐adjacent quadrupoles independently from each other. However, the PCB, the image acquisition software as well as the application of the actuators electric signal would need to be redesigned in order start the spectra acquisition of the cells in an overlapping timely manner.

*The authors have declared no conflict of interest*.

## Supporting information

Supporting Information

Supporting Information

Supporting Information

Supporting Information

Supporting Information

Supporting Information
