# Supplementary material for: On‐chip technology for single‐cell arraying, electrorotation‐based analysis and selective release
Source: Electrophoresis. 2019 Jun 3;40(14):1830–8. doi: 10.1002/elps.201900097 (PMC6771916; doi:10.1002/elps.201900097)
Supplement: Supplementary file 1 — Supporting Information [file ELPS-40-1830-s006.pdf]

On-chip technology for single-cell arraying, electrorotation-based analysis and selective release

Supplementary information

Kevin Keim, Mohamed Z. Rashed, Samuel C. Kilchenmann, Aurélien Delattre, António F. Gonçalves, Paul Éry and Carlotta Guiducci

Laboratory of Life Sciences Electronics – École Polytechnique Fédérale de Lausanne, Switzerland

1. Evaluation of the stability of the acquisition of electrorotation spectra

Experiments were performed to show the stability of the electrorotation system as well to verify the functionality of the proposed system.

A study on effect of changing the applied voltage on the speed of rotation was conducted. The electrorotation spectra of a single HEK 293 cell were acquired at different applied voltages, as shown in Supplementary Figure 1 (a). The speed of rotation increases quadratically with increasing applied voltage, as predicted by eq. 2 in the main manuscript.

Moreover, electrorotation spectra of a single HEK 293 cell were acquired every 5 minutes over a duration of 30 minutes while the cell was constantly kept rotating in an electric field. As can be seen from the spectra shown in Supplementary Figure 1 (b), the electrorotation spectrum remains stable over the whole duration of the experiment. Changes of the speed of rotation are of less than ±10% overall.

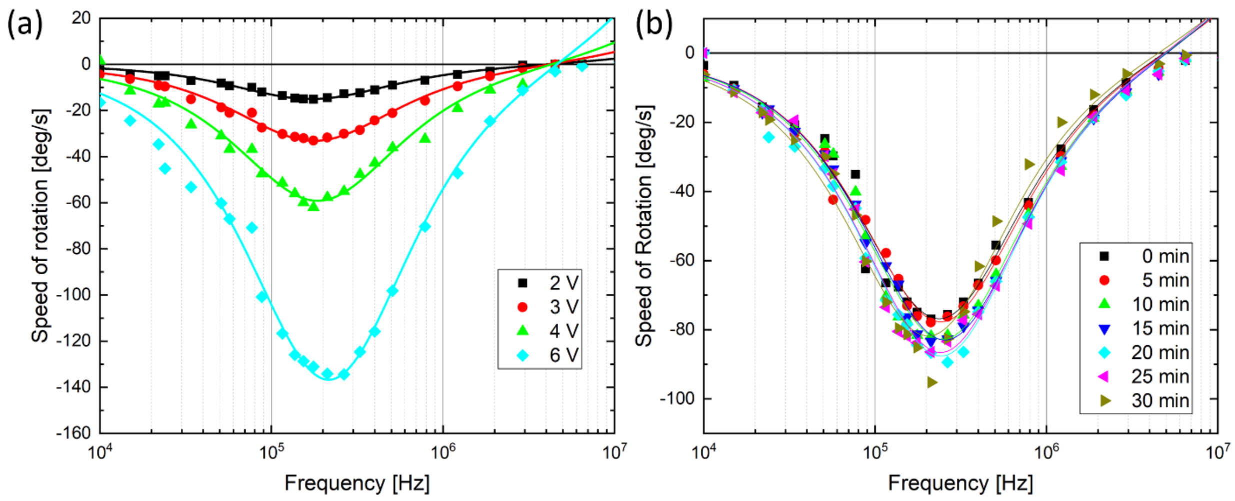

Supplementary Figure 1 (a) Non-normalized electrorotation spectra of a HEK 293 cell for different applied amplitude of the signal. (b) Non-normalized spectra of a single HEK 293 cell. 7 spectra were acquired subsequentially with 5 minutes interval for 30 minutes.

In the main manuscript we present averaged spectra of 17 T lymphocytes, 33 HEK 293, 14 HeLa and 29 M17 neuroblastoma. The data shown contains only the data of the first electrorotation spectrum of each cell. The original spectra of each cell was normalized by dividing it by the maximum speed of rotation as can be shown in Supplementary Figure 2.

Kevin Keim, Mohamed Z. Rashed, Samuel C. Kilchenmann, Aurélien Delattre, António F. Gonçalves, Paul Éry and Carlotta Guiducci

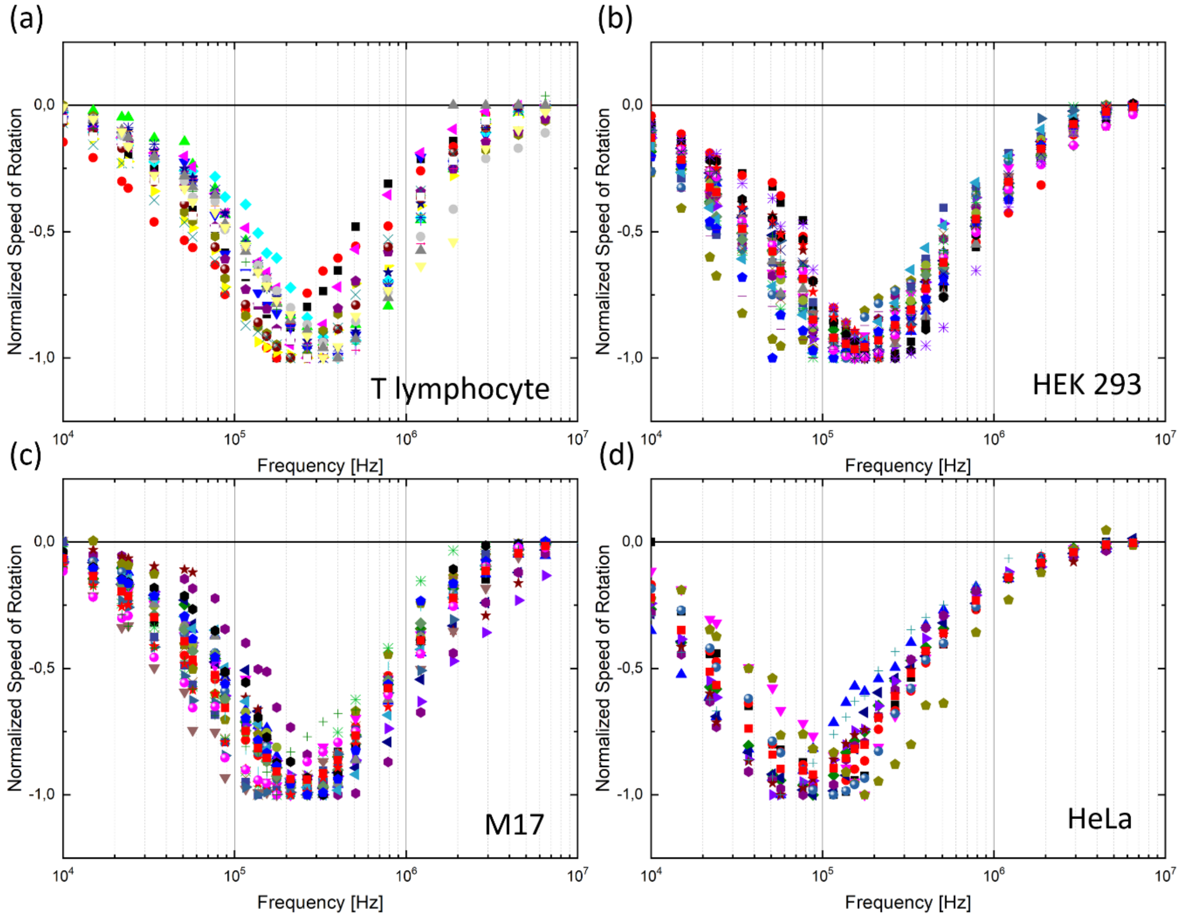

Supplementary Figure 2 Measured electrorotation spectra of (a) 20 immortalized human T lymphocytes, (b) 33 HEK 293 cells, (c) 29 M17 neuroblastoma cells, and (d) 14 HeLa cells. Normalized by the maximum rotation speed.

Kevin Keim, Mohamed Z. Rashed, Samuel C. Kilchenmann, Aurélien Delattre, António F. Gonçalves, Paul Éry and Carlotta Guiducci

2. Finite element simulations

The finite element simulations presented in the main manuscript were performed for all cell types (T lymphocytes, HEK 293, M17 and HeLa cells) at different medium conductivities (1 mS/m, 10 mS/m, 100 mS/m, and 1000 mS/m), different electric field frequencies (10 kHz, 100 kHz, 1 MHz and 10 MHz), different pressure differences (0.001 mbar, 0.01 mbar, 0.05 mbar and 0.1 mbar) and different electrode layouts (interelectrode distances of 20 µm, 40 µm and 80 µm). The trapping behavior of cells of the different configurations was investigated. It turns out that at conditions which are convenient for combined electrorotation and DEP trapping experiments (100 mS/m and 100 kHz DEP signal) HEK 293, M17 and HeLa cells are behaving very similar. They are being trapped at a medium pressure difference (0.01 mbar at an inter electrode of 80 µm and 0.05 mbar at an inter electrode of 80 µm), as shown in Supplementary Figure 3(e-h) and in the main manuscript. T lymphocytes in contrast are being slowed down, but are finally flowing through the traps. However, in smaller cell traps as with an inter electrode distance of 20 µm and a pressure difference of 0.05 mbar, T lymphocytes are getting trapped, while the other cell types are partially not overcoming the first dielectric barrier as shown in Supplementary Figure 3 (b-d).

The main reason for the different trapping behavior is related to the size of the cells, since the DEP force depends on the cell radius cube, as shown in eq. (1) in the main manuscript. T lymphocytes are the smallest cells investigated in this study, therefore, they are the hardest to trap.

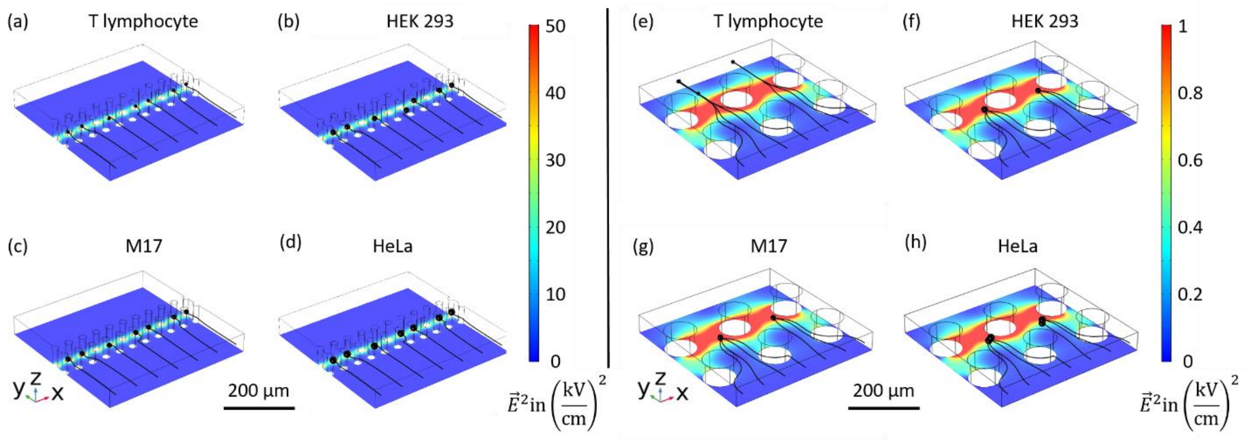

Supplementary Figure 3 Finite element simulations of the trajectory of different cell types in a microfluidic channel. The 3D electrodes in the middle of the microfluidic channel have a diameter and an interelectrode distance of 20 µm (a-d) and of 80 µm (e-h). The applied voltage at the exit electrodes is 5 V amplitude and at the entrance electrodes 1 V amplitude. The squared electric field in the microfluidic channel is illustrated in the rainbow colors. The cells trajectory in black illustrates that T lymphocytes are (a) getting trapped within the electrode array of an inter electrode distance of 20 µm, while HEK 293, M17 and HeLa cells (b-d) partially blocked by the lower entrance barrier and do not enter the micro cages In an array of an inter electrode distance of 80 µm T lymphocytes are not getting trapped (e), while the other cells are getting trapped (f-h).

3. Printed Circuit Board

A printed circuit board (PCB) was designed in order to address every electrode on the chip separately. The PCB has a whole cut in the middle to facilitate the vision of the chip under inverted microscope as shown in Supplementary Figure 4.

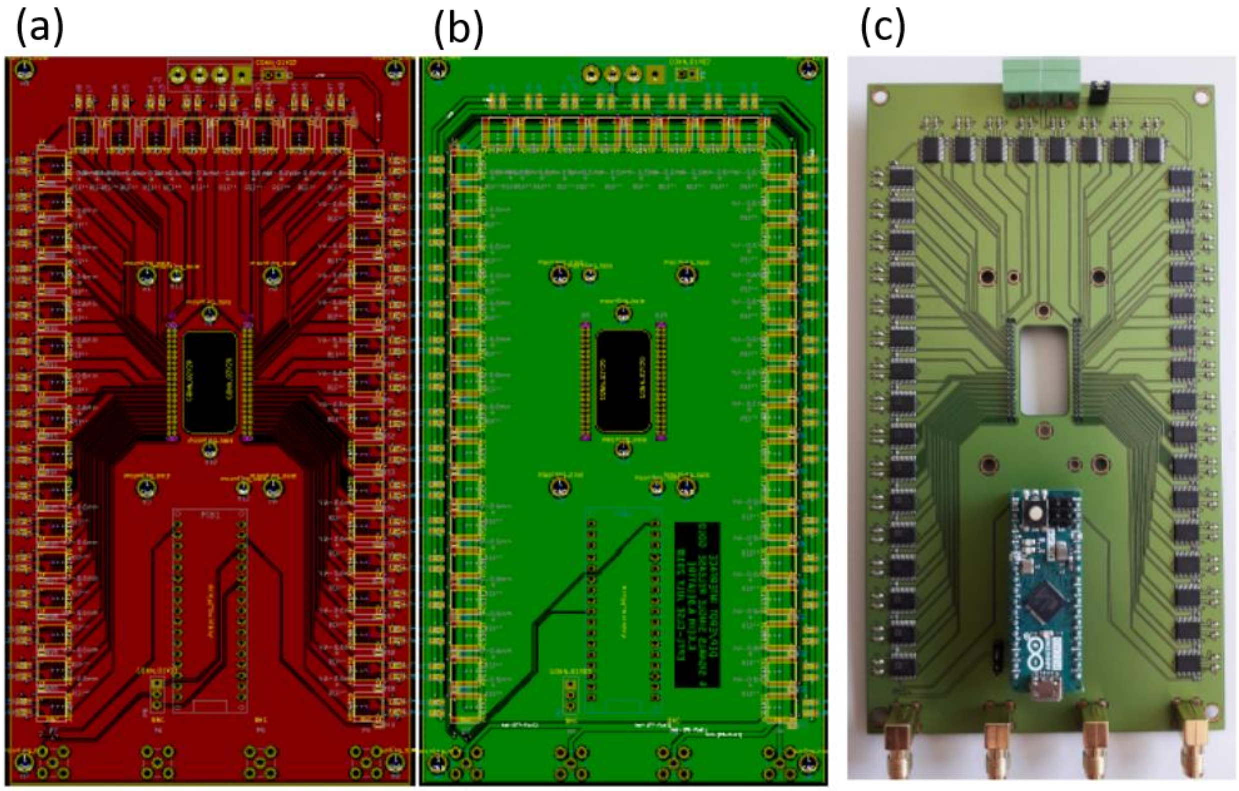

Supplementary Figure 4 Top views of the PCB. (a) Copper mask for the PCB showining the connection lines from the springloaded contacts on the left and right sides of the cut hole connecting the electrodes to the multiplexers that are selectively switched in order to address a specific elctrode. (b) The connections lines of the bottom part of the PCB from where the four signals supplied from the frequency generator through the SMB connectors- at the bottom of the PCB- to the multiplexers (c) Photograph of the final fabricated and assembled PCB. Connection to the frequency generator are achieved through the four SMB connectors at the bottom. The power is supplied by the green connector at the top of the PCB. The Arduino controller is placed central below the cut hole for the chip observation and is connected via mini USB to the controlling computer.

On the left and the right side of the hole cut, 40 spring loaded contacts are placed to connect each electrode on the chip individually to the PCB. Etched copper lines shown in Supplementary Figure 4 (a) are connecting each contact to an exit of an ADG1439 multiplexer.

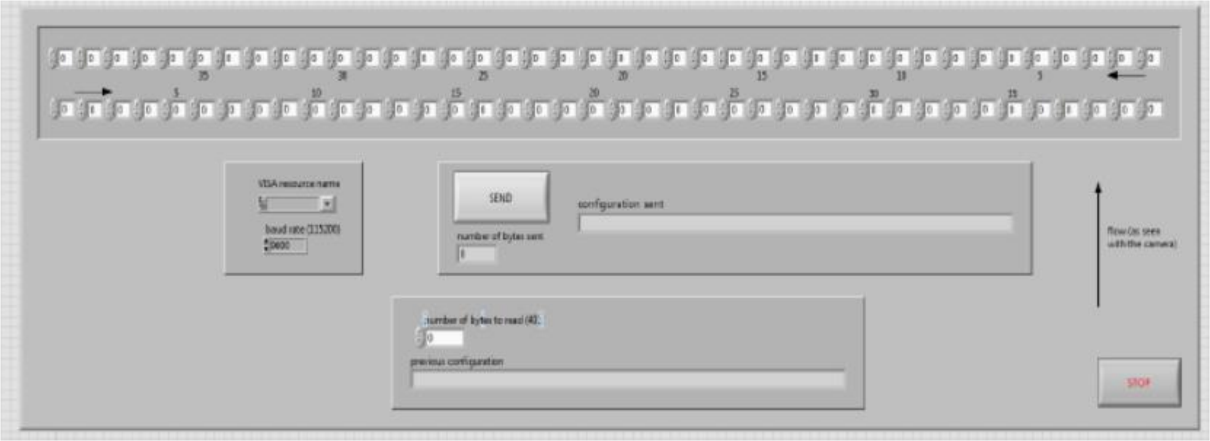

Supplementary Figure 5 Control interface of the LabView program controlling the multiplexers. The operator can address any electrode by selecting the signal type 1,2,3,4 or 0 (0 corresponds to no signal applied) from a selection menu. By clicking send, the new signal is updated by the multiplexers. For electrorotation experiments a circular/anticircular order for one quadrupole of electrodes has to be chosen.

Each multiplexer can route four different input signals (or no signal) to two outputs. Therefore, by such MUX we can decide which of the four signals supplied by the frequency generator is routed to which electrode, or if there is no signal at all applied (OFF state). The four signals from the frequency generator are connected to the PCB by four SMB connectors as shown at the bottom of Supplementary Figure 4 (c). Each signal line supplies every multiplexer with all four signals. In Supplementary Figure 4 (b), below the hole cut for observation, the wiring for the Arduino (microcontroller) is shown. Two lines connect all ADG1439 in series. One line is used to provide the clock signal, which gives the timing for the switches and the other line is the control line, which ultimately passes the signal from the Arduino to the switches. The Arduino is controlled by a LabView interface, as shown in Supplementary Figure 5.

4. Field of vision and parallelized acquisition of electrorotation spectra

As discussed in the main manuscript, the field of vision with a 20X magnification is 690 µm × 582 µm and therefore 10 microcages of an electrode array of a 40 µm inter electrode distance can be observed under the microscope as shown in Supplementary Figure 6.

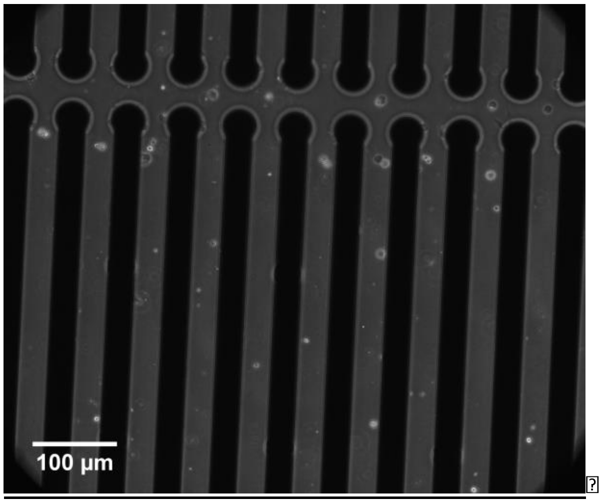

Supplementary Figure 6 Field of vision on an electrode array of 40 µm inter electrode distance with HeLa cells flowing inside the microchannel, using a 20x magnification. The field of vision with this magnification is 690 µm × 582µm and 10 quadrupoles can be visualized.

Electrorotation spectra of multiple single cells can be acquired simultaneously as shown for: three M17 neuroblastoma cells (Supplementary Figure 7); 3 human immortalized T lymphocytes (Supplementary Figure 8) and a HeLa cell and a M17 neuroblastoma cell (Supplementary Figure 9).

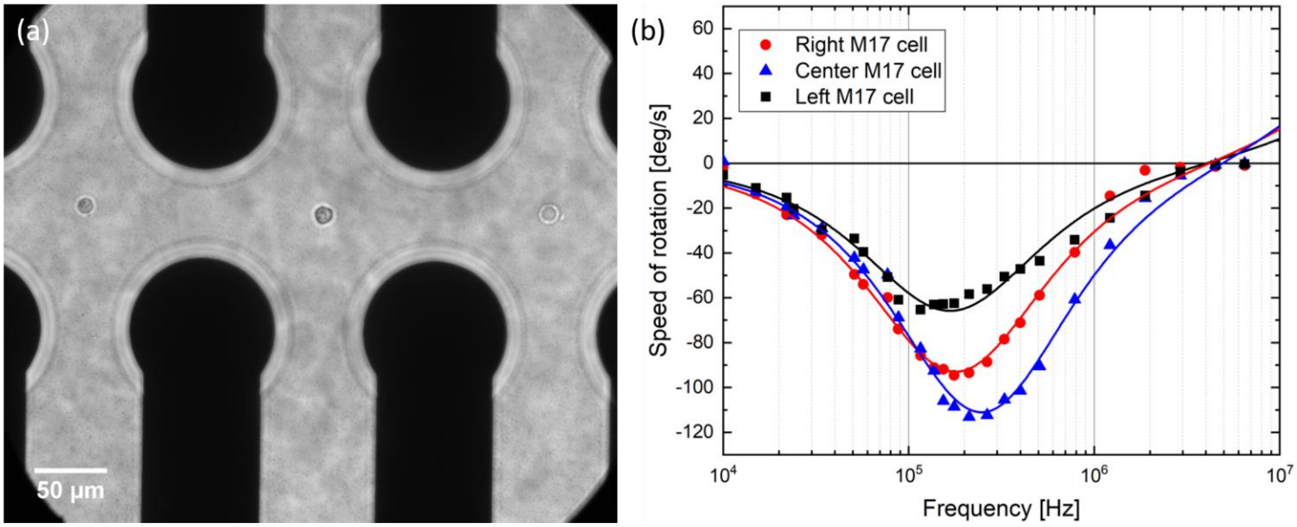

Supplementary Figure 7 (a) Three M17 neuroblastoma cells simultaneously rotating in three individual neighboring quadrupole cages of the array. (b) the corresponding electrorotation spectra of the three cells shown in (a).

On-chip technology for single-cell arraying, electrorotation-based analysis and selective release
Supplementary Information
Kevin Keim, Mohamed Z. Rashed, Samuel C. Kilchenmann, Aurélien Delattre, António F. Gonçalves, Paul Éry and Carlotta Guiducci

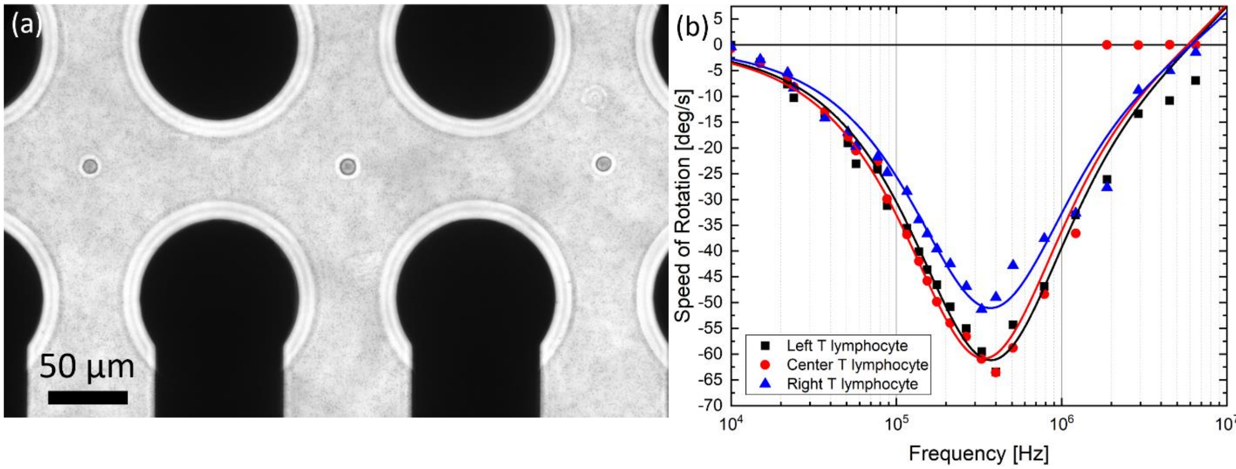

Supplementary Figure 8 (a) Three human immortalized T lymphocytes simultaneously rotating in three separate electrode cages of the array. (b) the corresponding electrorotation spectra of the Three cells shown in (a).

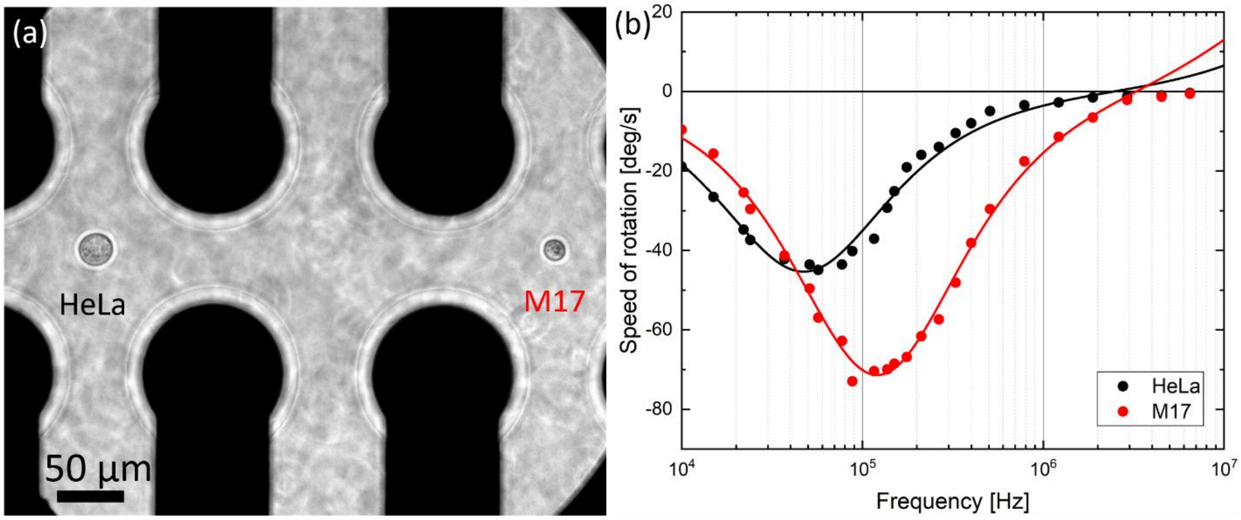

Supplementary Figure 9 (a) HeLa cell and M17 neuroblastoma cells simultaneously trapped and rotating in two individual cages of the array. (b) corresponding electrorotation spectra of the two cells

5. Bibliography

[1] J. Voldman, M. Toner, M. L. Gray, and M. A. Schmidt, "Design and analysis of extruded quadrupolar dielectrophoretic traps," *J. Electrostat.*, vol. 57, no. 1, pp. 69–90, 2003.

[2] L. Wang, L. Flanagan, and A. P. Lee, "Side-wall vertical electrodes for lateral field microfluidic applications," *J. Microelectromechanical Syst.*, vol. 16, no. 2, pp. 454–461, 2007.

[3] Y. K. Cho, T. H. Kim, and J. G. Lee, "On-chip concentration of bacteria using a 3D dielectrophoretic chip and subsequent laser-based DNA extraction in the same chip," *J. Micromechanics Microengineering*, vol. 20, no. 6, 2010.

[4] J. Xu *et al.*, "Electrofluidics fabricated by space-selective metallization in glass microfluidic structures using femtosecond laser direct writing," *Lab Chip*, vol. 13, no. 23, p. 4608, 2013.

[5] Chunlei Wang, Guangyao Jia, L. H. Taherabadi, and M. J. Madou, "A novel method for the fabrication of high-aspect ratio C-MEMS structures," *J. Microelectromechanical Syst.*, vol. 14, no. 2, pp. 348–358, Apr. 2005.

[6] R. Martinez-Duarte, R. A. Gorkin, K. Abi-Samra, and M. J. Madou, "The integration of 3D carbon-electrode dielectrophoresis on a CD-like centrifugal microfluidic platform," *Lab Chip*, vol. 10, no. 8, pp. 1030–1043, 2010.

[7] J.-W. Choi, S. Rosset, M. Niklaus, J. R. Adleman, H. Shea, and D. Psaltis, "3-Dimensional Electrode Patterning Within a Microfluidic Channel Using Metal Ion Implantation," *Lab Chip*, vol. 10, no. 6, p. 783, 2010.

[8] F. E. H. Tay, L. Yu, A. J. Pang, and C. Iliescu, "Electrical and thermal characterization of a dielectrophoretic chip with 3D electrodes for cells manipulation," *Electrochim. Acta*, vol. 52, no. 8 SPEC. ISS., pp. 2862–2868, 2007.

[9] C. Iliescu, G. L. Xu, V. Samper, and F. E. H. Tay, "Fabrication of a dielectrophoretic chip with 3D silicon electrodes," *J. Micromechanics Microengineering*, vol. 15, no. 3, pp. 494–500, 2005.

[10] S. Rajaraman *et al.*, "Microfabrication technologies for a coupled three-dimensional microelectrode, microfluidic array," *J. Micromechanics Microengineering*, vol. 17, no. 1, pp. 163–171, 2007.

[11] S. C. Kilchenmann, E. Rollo, E. Bianchi, and C. Guiducci, "Metal-coated silicon micropillars for freestanding 3D-electrode arrays in microchannels," *Sensors Actuators B Chem.*, vol. 185, pp. 713–719, Aug. 2013.

[12] S. C. Kilchenmann, E. Rollo, P. Maoddi, and C. Guiducci, "Metal-Coated SU-8 Structures for High-Density 3-D Microelectrode Arrays," *Journal of Microelectromechanical Systems*, vol. 25, no. 3, pp. 425–431, Jun-2016.

[13] K. Keim, A. Gonçalves, and C. Guiducci, "Trapping of Single-Cells Within 3D Electrokinetic Cages," in *Proceedings of the 2018 COMSOL Conference in Lausanne*, 2018.
